# Usefulness of a Nanostructured Fibrin-Agarose Bone Substitute in a Model of Severely Critical Mandible Bone Defect

**DOI:** 10.3390/polym13223939

**Published:** 2021-11-15

**Authors:** Miguel-Angel Martin-Piedra, Belén Gironés-Camarasa, Antonio España-López, Ricardo Fernández-Valadés Gámez, Cristina Blanco-Elices, Ingrid Garzón, Miguel Alaminos, Ricardo Fernández-Valadés

**Affiliations:** 1 Tissue Engineering Group, Department of Histology, Faculty of Medicine, University of Granada, E18016 Granada, Spain; mmartin@ugr.es (M.-A.M.-P.); cblanco@ugr.es (C.B.-E.); igarzon@ugr.es (I.G.); 2 Instituto de Investigación Biosanitaria ibs.GRANADA, E18012 Granada, Spain; 3 Division of Pediatric Surgery, University Hospital Virgen de las Nieves, E18014 Granada, Spain; belengironescamarasa@hotmail.com; 4 Doctoral Program in Biomedicine, University of Granada, E18071 Granada, Spain; 5 Craniofacial Malformations and Cleft Lip and Palate Management Unit, University Hospital Virgen de las Nieves, E18014 Granada, Spain; ajep@us.es; 6 Division of Oral and Maxillofacial Surgery, San Pedro Hospital, La Rioja, E26006 Logroño, Spain; rfvgamez@gmail.com

**Keywords:** bone, tissue engineering, fibrin-agarose, mandible, regeneration

## Abstract

Critical defects of the mandibular bone are very difficult to manage with currently available materials and technology. In the present work, we generated acellular and cellular substitutes for human bone by tissue engineering using nanostructured fibrin-agarose biomaterials, with and without adipose-tissue-derived mesenchymal stem cells differentiated to the osteogenic lineage using inductive media. Then, these substitutes were evaluated in an immunodeficient animal model of severely critical mandibular bone damage in order to assess the potential of the bioartificial tissues to enable bone regeneration. The results showed that the use of a cellular bone substitute was associated with a morpho-functional improvement of maxillofacial structures as compared to negative controls. Analysis of the defect site showed that none of the study groups fully succeeded in generating dense bone tissue at the regeneration area. However, the use of a cellular substitute was able to improve the density of the regenerated tissue (as determined via CT radiodensity) and form isolated islands of bone and cartilage. Histologically, the regenerated bone islands were comparable to control bone for alizarin red and versican staining, and superior to control bone for toluidine blue and osteocalcin in animals grafted with the cellular substitute. Although these results are preliminary, cellular fibrin-agarose bone substitutes show preliminary signs of usefulness in this animal model of severely critical mandibular bone defect.

## 1. Introduction

Several conditions and diseases may significantly affect the oral and maxillofacial region, including congenital malformations, trauma, infection, tumors, osteonecrosis, and other relevant pathologies [1] that can lead to significant loss of bone tissue [2]. However, large critical-size defects affecting the mandible are very difficult to manage, due to the unique histological and physiological characteristics of this bone [3]. In most cases, defects are repaired using bone grafts that can be obtained autologously [4] or from cadaveric donors [5,6], with variable results, although numerous inert biomaterials have also been tested. Specifically, several types of biomaterials based on calcium phosphate bioformulations—especially tricalcium phosphate and hydroxyapatite—have been extensively used for the reconstruction of critical mandibular defects, with promising results [7,8,9], although none of these biomaterials was fully efficient as an inductor of bone regeneration [10].

In general, reports related to the treatment of large critical-size bone defects are rare, as these conditions are very difficult to treat, and results are typically suboptimal [10]. A recent review of the literature related to critical mandibular bone defects demonstrated that the currently available evidence is very heterogeneous in terms of the animal models, defect size, follow-up time, and biomaterials used to treat the bone defects [10]. Promising results were described in rat calvarial defects using different types of biomaterials—such as hydroxyapatite combined with poly-lactic-co-glycolic acid [11], or composites containing wollastonite and β-tricalcium phosphate [12]—especially when biomaterials were combined with bone morphogenetic proteins (BMPs) [13]. In the human mandible, large critical defects were treated with an allogeneic bone matrix impregnated in BMP combined with a titanium mesh, but bone induction was only found only in one-third of the patients [14]. Overall, further research is needed in order to elucidate the usefulness of biomaterials used in large-defect bone tissue engineering [13].

An ideal graft should be highly biocompatible and able to induce bone regeneration without generating any significant side effects on the host, and it should allow host cells to remodel the graft and replace it with newly formed bone tissue [6,15]. However, the biocompatibility and regenerative potential of most currently available biomaterials is limited, and novel types of grafts capable of inducing effective bone regeneration are needed. One of the possible alternatives is the use of organic biomaterials based on natural components—such as human fibrin—which are known to have high biocompatibility, and have been successfully used in multiple regenerative applications, such as for the human cornea [16,17], oral mucosa [18], nerves [19], and skin [20,21]. Another possibility is the use of cells immersed in the biomaterial, since previous reports have demonstrated that the combination of biocompatible biomaterials with living cells is associated with a significant improvement in results in terms of the formation of bone tissue [10]—especially when stem cells are used [22]. Interestingly, the use of adipose tissue mesenchymal stem cells (ADSCs) pre-differentiated ex vivo to the osteogenic lineage was shown to increase the regenerative potential of several biomaterials as compared to native ADSC [23].

In the present work, we evaluated the bone regeneration potential of nanostructured fibrin-agarose biomaterials and fibrin-agarose biomaterials containing human ADSC differentiated to the osteogenic lineage in a model of severely critical bone defects of the rat mandible, and we determined their potential usefulness as biomaterials for bone repair.

## 2. Materials and Methods

### 2.1. Generation of Acellular and Cellular Bone Substitutes by Tissue Engineering

Acellular bone substitutes were generated in the laboratory using nanostructured fibrin-agarose biomaterials. Briefly, to generate 1 mL of hydrogel, 760 µL of human plasma obtained from plasma donors was mixed with 75 μL of DMEM (Dulbecco’s modified Eagle’s medium, Merck Life Science, Darmstadt, Germany), 15 µL of tranexamic acid as an antifibrinolytic agent (Amchafibrin 5 mg/mL, MEDA Pharma SL, Madrid, Spain) and 50 μL of a 2% solution of type VII agarose (Merck Life Science) dissolved in PBS. Then, 100 μL of 1% CaCl_2_ (Merck Life Science) was added in order to trigger the fibrin polymerization reaction. This mixture was aliquoted in 6-well plates and allowed to jellify in a cell culture incubator. 24 h later, hydrogels were carefully extracted from the culture plates and subjected to plastic compression nanostructuration to obtain a thin, consistent layer of biomaterial, as previously described [24]. Then, this structure was wound on itself to generate a consistent, multilayered, rod-shaped cylinder [19].

To generate cellular bone grafts—considered as human tissue-engineered bone substitutes—we first obtained human adipose tissue mesenchymal stem cell (ADSC) cultures. These cultures were established from small adipose tissue biopsies harvested from healthy donors subjected to programmed surgery, and digested with 0.3% type I collagenase (Gibco BRL Life Technologies, Waltham, MA, USA) at 37 °C. Then, 50,000 ADSCs were obtained and mixed with the fibrin-agarose mixture described above before inducing the hydrogel polymerization, and biomaterials were aliquoted and allowed to jellify in a cell culture incubator. Hydrogels were incubated for 21 days in an osteogenic induction medium composed of basal DMEM medium (Merck Life Science) supplemented with 10% fetal bovine serum (FBS, Sigma-Aldrich Inc. St. Luis, MO, USA), 1% antibiotic–antimycotic (100 U/mL penicillin G, 100 mg/mL streptomycin and 0.25 mg/mL amphotericin B), and different growth factors and inductive reagents (100 nM dexamethasone, 10 mM b-glycerol phosphate, and 50 mM L-ascorbic acid), as previously reported [25]. Finally, these biomaterials were subjected to nanostructuration and rolled up as described for the acellular grafts.

This study was conducted in accordance with the guidelines of the Declaration of Helsinki and approved by the Institutional Ethics Committee of the Province of Granada for research with human samples (Comité Ético de Investigación, CEIM/CEI), ref. 0018-N-19, and informed consent was obtained from tissue donors.

### 2.2. Analysis of Biomechanical Properties

The biomechanical properties of the fibrin-agarose bioartificial tissues generated in the present work were evaluated using an Instron Model 5943 biomechanical analyzer (Norwood, MA, USA) with Bluehill 3 software. Bioartificial tissues were subjected to nanostructuration and rolled up as described above, and rod-shaped cylinders were placed on the holding clamps of the device, leaving a distance of 1 cm between both clamps. The biomechanical analyzer was programmed to run with a strain rate of 5 mm/min of continuous traction, until rupture of the sample. The following parameters were analyzed in each sample using the Instron Bluehill 2 materials testing software: Young’s modulus, stress at fracture, break load, and strain at fracture. Five samples were analyzed (*n* = 5).

### 2.3. Animal Models

**Figure 1:**
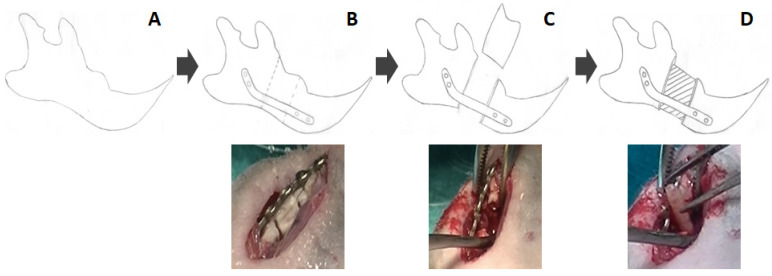
Generation of a severely critical-size bone defect in Foxn1rnu nude rats: (**A**) The mandibular body was surgically exposed. (**B**) A titanium microplate was fixed, and a 1-cm-long bone segment was sectioned. (**C**) The sectioned bone segment was excised. (**D**): A tissue-engineered bone substitute was implanted at the defect site.

To evaluate the efficiency of each bone graft in inducing bone tissue regeneration, acellular and cellular grafts were implanted at the defect site of Foxn1rnu nude rats in which a severely critical bone defect was generated at the right side of the mandible (Figure 1). The left side was left untouched and used as a control in each animal. Four study groups were included in the present work:

(1) P-CTR group (*n* = 2): Native normal animals used as positive controls. These animals were not subjected to any surgical procedures;

(2) N-CTR group (*n* = 4): Animals subjected to surgical removal of a fragment of the right mandible, used as negative controls. First, animals were deeply anesthetized with ketamine and acepromazine (Boehringer Ingelheim, Ingelheim am Rhein, Germany). The base of the mandibular body was then surgically exposed, and a 1-cm-long fragment was sectioned and extracted using a bone saw. To stabilize the mandible and enable mandibular function, a titanium microplate was previously fixed with 2 screws at each side of the defect. Finally, the soft tissues were repaired, and the skin incision was closed using surgical suture material;

(3) ACELL group (*n* = 4): Animals subjected to surgical removal of a fragment of the right mandible, as described for the N-CTR group, but an acellular bone graft generated as described above was implanted at the defect site;

(4) HTEB group (*n* = 8): Animals subjected to surgical removal of a fragment of the right mandible, with a cellular bone graft (human tissue-engineered bone substitute) generated as described above implanted at the defect site.

In all animals subjected to surgical procedures, analgesia was used for 7 days after surgery, and soft rat chow was provided to the animals to facilitate chewing. All animals were kept at the animal facility of the University Hospital Virgen de las Nieves and Instituto de Investigación Biosanitaria ibs.Granada, Spain, under veterinary supervision. Rats were euthanatized after 4 months of follow-up.

Animal experimentation was approved by the animal ethics and research committee of the University of Granada (CEEA) and the Regional Ministry of Agriculture (Consejería de Agricultura, Ganadería, Pesca y Desarrollo Sostenible), Junta de Andalucía, Spain (ref. 08/07/2019/122).

### 2.4. CT Scan Analysis of Cranial Bone Structure and Morphology

Immediately after euthanasia, animals were analyzed using a Pointnix Point 3D Combi 500 CT scanner, as previously reported [26]. For this purpose, the head of each animal was placed and fixed on the analysis surface, and high-resolution images were obtained from all cranial structures. For each animal, 3D reconstruction images were obtained, and the following variables were assessed:

(1) Morphological analysis of cranial structure and symmetry. The 3D reconstruction images were evaluated by three independent researchers, and teeth morphology, bite function (as determined by dental occlusion), and facial symmetry were evaluated as normal or pathological;

(2) CT radiodensity of the regeneration tissue. For each animal, axial plane tomographic sections were obtained at the site of the defect at the right side of the mandible, also including the control left mandible. Images were then analyzed by three independent researchers to qualitatively determine the presence of regeneration tissue at the site of the defect. Then, this regeneration tissue was quantitatively analyzed by measuring its radiodensity as determined by Hounsfield units (HU). Six independent points were randomly selected within the regeneration site at the right side of the mandible, or its counterpart at the left side of the mandible and in control animals, and radiodensity was automatically calculated by the software of the equipment.

### 2.5. Histological Analysis

For histological analysis, the mandible of each animal was surgically dissected and fixed for 24 h in 10% formalin. Tissues were then decalcified using Anna Morse reagent consisting of 50% formic acid and 20% sodium citrate (both from Panreac Química S.L.U., Barcelona, Spain) until the bone became soft (around 5–6 days). Decalcified tissues were sectioned using a surgical blade, and the defect site was photographed in order to macroscopically evaluate the presence of regeneration tissue at the defect site. Tissues were then dehydrated and embedded in paraffin using routine methods, and tissue sections were obtained with a microtome and mounted on glass slides.

For histological analysis, tissue sections were dewaxed, rehydrated, and stained with hematoxylin and eosin (H&E) following standard protocols. Images were obtained using a Nikon Eclipse 90i light microscope (Nikon Corp., Tokyo, Japan). In each sample, the site of the defect on the right side of the mandible and the equivalent site on the control left side were analyzed.

To evaluate the presence of relevant components of the extracellular matrix (ECM) at the site of the defect and in the control mandibular bone, tissue samples were subjected to the following histochemical methods [27]: calcium deposits and mineralization were identified with alizarin red; collagen fibers were stained and evaluated with picrosirius red; and proteoglycans were stained with alcian blue and toluidine blue histochemistry. Analysis of the bone marker osteocalcin and the ECM component versican was performed via immunohistochemistry. With this purpose, samples were subjected to antigen retrieval using pH 6 citrate buffer (0.01 M) at 98 °C for 5 min, and endogenous peroxidase was quenched with 3% H_2_O_2_ (Panreac Química S.L.U.). After prehybridization, sections were incubated at 37 °C with proteinase-k (ready-to-use solution, Agilent Dako, Santa Clara, CA, USA) and chondroitinase ABC from *Proteus vulgaris* (0.2 U/mL, Merck Life Science) for 30 min for osteocalcin and versican, respectively. Then, samples were incubated overnight with a 1:200 solution of anti-osteocalcin primary antibody (Abcam ref. 13420, Cambridge, UK) and 1:100 of anti-versican (Abcam ref. 19345), washed, and incubated with a 1:500 solution of secondary anti-rabbit antibody for 30 min. A 3,3′-diaminobenzidine solution (Vector Laboratories, Burlingame, CA, USA) was used to reveal the positive signal, and tissues were counterstained with Mayer’s hematoxylin and mounted using glass coverslips.

To determine the ECM composition in the different samples, the histochemical and immunohistochemical results found for osteocalcin and versican were quantified by measuring the ECM signal intensity using ImageJ multipoint analysis tools (National Institutes of Health, Bethesda, MD, USA), as previously described [28]. For each sample, 10 dots were randomly selected at the regeneration site at the right mandible (or at its counterpart in normal bone), and the signal intensity was automatically calculated by the program. In cases in which areas of ossification or chondrification were found at the regeneration area, these areas were quantified independently. Quantification analyses were carried out by blinded researchers.

### 2.6. Statistical Analysis

In order to identify statistical differences within the study group, quantification results obtained for regeneration tissue radiodensity (in HU) and histochemical and immunohistochemical analysis (quantified as signal intensity) were statistically analyzed. First, variables were evaluated with the Shapiro–Wilk test to determine whether they were normally distributed. As this test demonstrated that the distributions were not normal, we used nonparametric statistics.

For radiodensity and histology, we compared the results obtained at the defect site at the right side of the mandible of each group of animals with results corresponding to the same area at the right side of the mandible of P-CTR (normal bone), and with results obtained at this area in N-CTR. For radiodensity, values obtained at the defect site at the right mandible were compared with those obtained at the homologous site at the left mandible in the same group of animals (used as controls).

All of these comparisons were carried out using pairwise Mann–Whitney exact tests. Data were statistically evaluated using RealStatistics (Dr. Charles Zaiontz, Purdue University, West Lafayette, IN, USA), and *p*-values below 0.05 were considered to be statistically significant using double-tailed tests.

## 3. Results

### 3.1. Biomechanical Testing

Analysis of the biomechanical properties of the fibrin-agarose bioartificial tissues generated in the present work showed that the average Young’s modulus of the analyzed samples was 0.49 ± 0.0 MPa, whereas the stress at fracture was 0.99 ± 0.2 MPa, the break load was 0.77 ± 0.1 N, and the strain at fracture was 28.77 ± 9.1 mm.

### 3.2. Morphological Analysis of Cranial Structure and Symmetry

**Figure 2:**
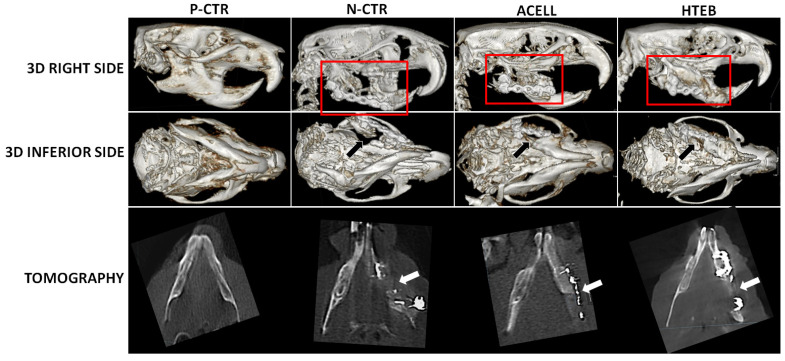
CT scan analysis of the head bones of each group of animals included in the study. Top panels correspond to the 3D reconstruction observed from the right side or from the inferior side, whereas bottom panels show a tomographic section obtained at the axial plane at the site of the defect. P-CTR: native normal animals, used as positive controls; N-CTR: animals subjected to surgical removal of a fragment of the right mandible, used as negative controls; ACELL: animals subjected to surgical removal of a fragment of the right mandible and implant of a nanostructured fibrin-agarose biomaterial; HTEB: animals subjected to surgical removal of a fragment of the right mandible and implant of a nanostructured human tissue-engineered bone substitute.

As shown in Figure 2, morphological analysis of the animals included in the study revealed several differences between groups. When P-CTR animals (native rats) were analyzed, we found that the morphology of the teeth was normal, allowing a physiological bite function, and the animals’ faces were symmetrical. In contrast, N-CTR animals showed several morphological alterations, with 75% of the animals showing teeth alterations (mainly, overgrowth and displacement of the superior incisors) associated with bite abnormalities in 50% of the cases (mainly, mandible retraction and tooth malocclusion), and facial asymmetry in 75% of the animals. When animals grafted with an acellular biomaterial (ACELL group) were analyzed, we found that 75% of the animals had normal teeth and were free from detectable bite alterations, and none of the animals showed facial asymmetry. Finally, animals grafted with a complete bone substitute (HTEB group) showed that 62.5% of the animals showed normal tooth morphology, and 87.5% had normal bite and normal facial symmetry.

### 3.3. Analysis of the Defect Site

**Figure 3:**
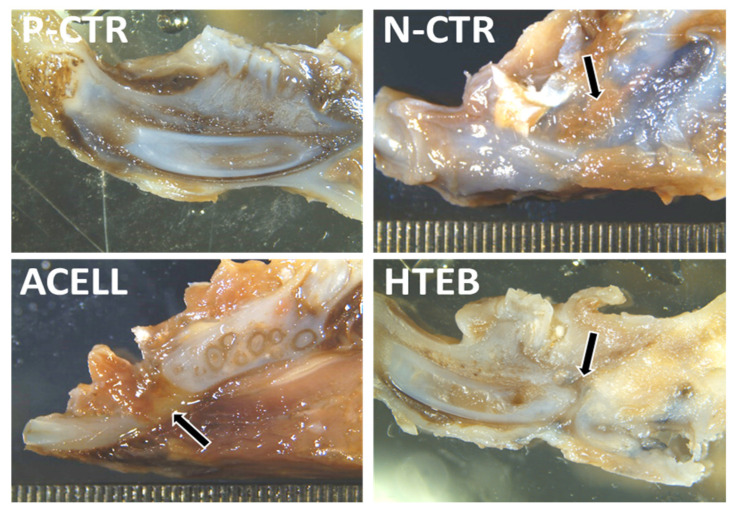
Gross macroscopic view of the right mandible of each group of animals included in the study. Tissues were fixed, decalcified, and sectioned in the sagittal plane to show the area of the defect. P-CTR: native normal animals, used as positive controls; N-CTR: animals subjected to surgical removal of a fragment of the right mandible, used as negative controls; ACELL: animals subjected to surgical removal of a fragment of the right mandible and implant of a nanostructured fibrin-agarose biomaterial; HTEB: animals subjected to surgical removal of a fragment of the right mandible and implant of a nanostructured human tissue-engineered bone substitute.

In order to assess the regeneration tissue generated at the defect site, we first analyzed the site of the mandibular defect in decalcified tissues corresponding to each group of animals. Macroscopic results (Figure 3) showed that the mandibles of positive control animals had a homogeneous structure with no detectable defects, while N-CTR animals displayed an area of soft tissue between both bone fragments at the defect site. ACELL animals also showed abundant soft tissue at the implant site, whereas the soft tissue found between both bone ends tended to be smaller and less abundant in HTEB animals.

Next, we evaluated the presence of bone at the defect site in CT images. As shown in Figure 2, the mandibular structure was regular and homogenous in positive controls, as expected. However, N-CTR animals displayed a large defect underneath the mandibular fixation plate, which was evident in the 3D reconstruction images, and showed the absence of dense tissue formation at the regeneration site in the tomography images, suggesting a complete absence of bone regeneration in this group of animals. For ACELL animals, a defect was also found at the regeneration site, although the 3D reconstruction images suggest that this defect could be smaller in ACELL animals as compared to N-CTR. Interestingly, tomography images suggest the presence of a regeneration tissue at the implant area, although the radiodensity of this tissue was very low in these animals. Finally, the 3D reconstruction images of HTEB animals revealed the presence of a regenerative tissue partially filling the defect site, although its consistency was not comparable to bone tissue in tomography images, suggesting that this tissue could partially correspond to soft tissue.

Quantitative analysis of the CT radiodensity of the tissue found at the defect site at the right side of the mandible revealed significant differences between groups. Results showed that positive controls showed 2742.8 ± 1171 Hounsfield units (HU) at the right mandible, whereas N-CTR had 546.7 ± 130.7 HU, ACELL had 542.4 ± 135.4 HU, and HTEB showed 884.3 ± 358.2 HU at the defect site. Statistical comparison between groups demonstrated that the CT intensity signal of positive control animals was significantly higher than N-CTR, ACELL, and HTEB animals (*p* < 0.05), while that of HTEB was significantly higher than that of N-CTR and ACELL animals (*p* = 0.002). In addition, when the radiodensity was compared between the right and the left sides of each animal, we found that both sides were similar in positive control animals (*p* > 0.05), whereas significant differences were detected between the two sides of the mandible in the N-CTR, ACELL, and HTEB groups (*p* < 0.05).

### 3.4. Histological Analysis

**Figure 4:**
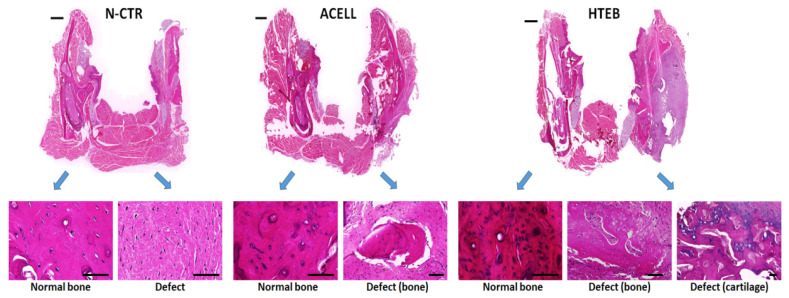
Histological analysis of mandible tissues using hematoxylin and eosin (H&E) staining. Normal bone: tissues corresponding to the non-operated side of the mandible (**left side**); defect: tissues corresponding to the operated side of the mandible (**right side**), showing some areas of bone and cartilage tissue found at this side; N-CTR: animals subjected to surgical removal of a fragment of the right mandible, used as negative controls; ACELL: animals subjected to surgical removal of a fragment of the right mandible and implant of a nanostructured fibrin-agarose biomaterial; HTEB: animals subjected to surgical removal of a fragment of the right mandible and implant of a nanostructured human tissue-engineered bone substitute. Scale bars correspond to 1000 µm in the top images representing low-magnification images and 50 µm in the images below obtained at higher magnifications.

Histological analysis of the non-operated mandibles (left side) of all animals included in the study—and both sides of P-CTR mandibles—using H&E showed that the bone structure was compatible with a normal compact bone consisting of a dense extracellular matrix with abundant osteoblasts and vascular lacunae forming osteons compatible with a normal Haversian bone. Then, analysis of the operated side of N-CTR animals showed a complete absence of bone tissue; instead, the area of defect was filled with a dense connective tissue consisting of a dense matrix with numerous fibroblast-like cells. For the operated mandibles of ACELL animals, we found that the defects were filled with a dense connective tissue similar to that found in N-CTR animals, but a few small, isolated areas of bone were found immersed within this connective tissue (average 1.25 ± 0.35 areas per animal). These areas were formed by normal bone with an osteoblast population immersed in a dense, well-organized extracellular matrix. Finally, analysis of HTEB animals showed that the defect zone was mostly composed of dense connective tissue, but a higher number of scattered bone areas was found (average 3 ± 1.41 areas). Interestingly, areas of developing cartilage were found close to some of these bone zones (Figure 4).

**Figure 5:**
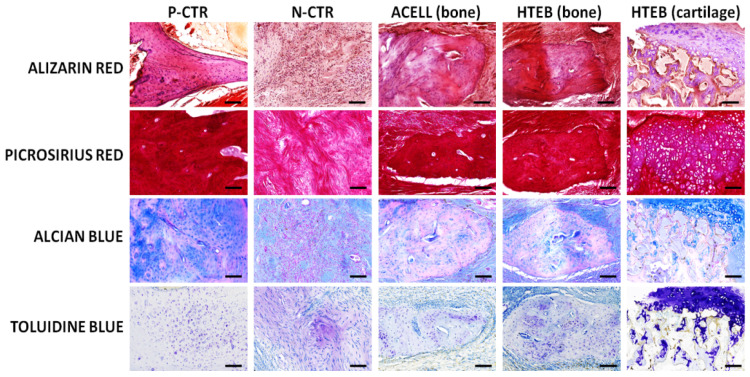
Analysis of ECM components at the defect area of each group of animals included in the study, as determined by histochemistry using alizarin red, picrosirius red, alcian blue, and toluidine blue. P-CTR: native normal animals, used as positive controls; N-CTR: animals subjected to surgical removal of a fragment of the right mandible, used as negative controls; ACELL: animals subjected to surgical removal of a fragment of the right mandible and implant of a nanostructured fibrin-agarose biomaterial; HTEB: animals subjected to surgical removal of a fragment of the right mandible and implant of a nanostructured human tissue-engineered bone substitute. Scale bars: 100 µm.

**Figure 6:**
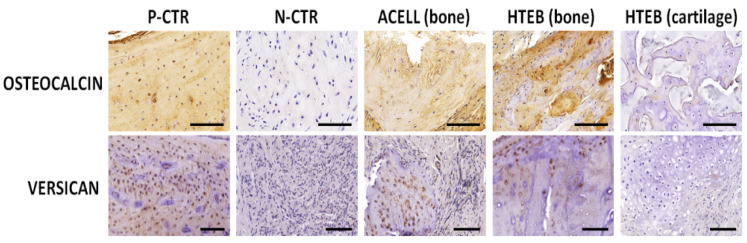
Analysis of ECM components at the defect area of each group of animals included in the study, as determined by immunohistochemistry for osteocalcin and versican. P-CTR: native normal animals, used as positive controls; N-CTR: animals subjected to surgical removal of a fragment of the right mandible, used as negative controls; ACELL: animals subjected to surgical removal of a fragment of the right mandible and implant of a nanostructured fibrin-agarose biomaterial; HTEB: animals subjected to surgical removal of a fragment of the right mandible and implant of a nanostructured human tissue-engineered bone substitute. Scale bars: 100 µm.

**Figure 7:**
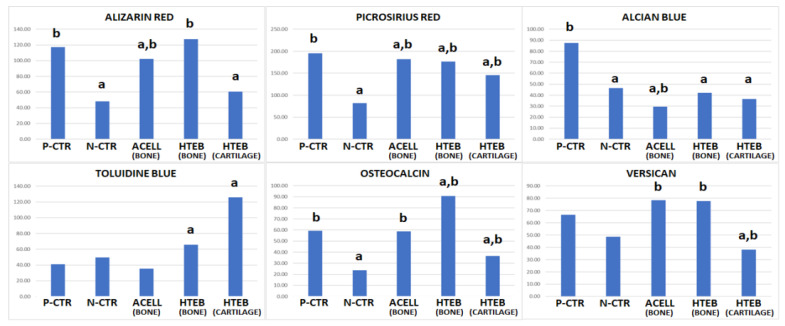
Quantitative analysis of ECM components as determined by histochemistry (alizarin red, picrosirius red, alcian blue, and toluidine blue) and immunohistochemistry (osteocalcin and versican). Results correspond to average values in each study group. P-CTR: native normal animals, used as positive controls; N-CTR: animals subjected to surgical removal of a fragment of the right mandible, used as negative controls; ACELL: animals subjected to surgical removal of a fragment of the right mandible and implant of a nanostructured fibrin-agarose biomaterial; HTEB: animals subjected to surgical removal of a fragment of the right mandible and implant of a nanostructured human tissue-engineered bone substitute. a: differences with P-CTR are statistically significant; b: differences with N-CTR are statistically significant.

In addition to the H&E analysis, we evaluated the implant area using several histochemical and immunohistochemical methods (Figure 5, Figure 6 and Figure 7). Initially, detection of calcification areas using alizarin red histochemistry revealed that the bone tissue found in P-CTR animals (normal controls) showed strong staining intensity, revealing the presence of abundant calcium deposits. In contrast, tissue found at the regeneration area of N-CTR animals contained very few alizarin-red-positive calcium deposits, with statistically significant differences from positive controls. Analysis of the areas of bone found at the regeneration area of ACELL animals showed lower staining intensity than positive controls, but contained higher amounts of calcium deposits than negative controls. However, we found that the staining intensity of bone areas corresponding to the HTEB group was similar to positive controls, and statistically higher than negative controls. Finally, cartilage areas found in HTEB animals showed very low alizarin red staining intensity (comparable to negative controls). Secondly, we used picrosirius red histochemistry to assess the presence of collagen fibers in each group of animals as a relevant component of the bone ECM. Our results showed that the highest collagen content was found in P-CTR normal bone, and the rest of the samples analyzed here displayed significantly lower picrosirius red intensity. The lowest levels of collagen were found at the regeneration area of N-CTR animals, with levels significantly lower than in ACELL and HTEB bone areas and HTEB cartilage areas.

Next, we analyzed the presence of ECM proteoglycans in each group of samples using alcian blue and toluidine blue histochemistry. For alcian blue, staining was significantly higher in P-CTR normal bone compared to the rest of the samples, and bone found in the ACELL group was significantly lower than N-CTR. No differences from N-CTR were found for HTEB bone and cartilage. Regarding toluidine blue, HTEB bone and cartilage showed significantly higher staining intensity than P-CTR, and no differences were found from N-CTR.

For the bone marker osteocalcin (Figure 6 and Figure 7), immunohistochemistry revealed strong staining intensity in P-CTR normal bone and a very low signal in N-CTR, with statistically significant differences between both groups of samples. Bone areas found at the regeneration sites of ACELL and HTEB animals showed significantly higher intensity than N-CTR, and bone found in HTEB animals had higher signal than P-CTR. Analysis of versican expression showed that the staining intensity was similar in the P-CTR, ACELL, and HTEB groups, whose signal intensity was significantly higher compared to N-CTR. The lowest signal was found in cartilage areas corresponding to HTEB samples.

## 4. Discussion

Induction of efficient and successful bone regeneration is a challenge in maxillofacial surgery. In the present work, we used a combination of biocompatible fibrin-agarose biomaterials with potential utility in tissue engineering [16,17,18,19,20], and pre-differentiated ADSCs that were previously demonstrated to have significant osteogenic potential [23]. Application of nanostructuration methods allowed us to generate three-dimensional cylinder-type structures that were used to repair the mandible defects generated in an animal model. Although the biomechanical properties of these bioartificial structures were lower than those of mineralized bone [29], we found that the biomechanical behavior of our tissue substitutes was high compared to previously reported values for fibrin-based hydrogels [30]. Most likely, application of the biofabrication methods described in the present work—including nanostructuration and modification of the three-dimensional structure of the biomaterial to generate a rod-shaped cylinder—was able to improve the biomechanical properties of this type of biomaterial, as previously suggested [31]. However, future biofabrication protocols should be developed to enhance these properties and make them more similar to those of native human bone.

In general, our results showed that the use of this biomaterial-based approach was not able to induce completely satisfactory bone regeneration in vivo. However, some positive results were obtained. There could be multiple reasons that our model was not able to induce a completely satisfactory bone regeneration process. First, it is possible that our model requires longer periods of time to achieve bone regeneration. In the present work, we analyzed our animals for 4 months after the surgical procedure, which is considered to be enough for a full regeneration in the rat, according to several reports [32]. However, the critical size of the defect induced here could require longer periods of time. Second, a crucial factor of the present work is the nature and size of the defect created in the mandibles of the study animals. Most previously reported works make use of a 3–5 mm circular defect in the inferior border of the rat mandible [33,34,35,36]. Although this defect is considered to be of critical size, the general structure of the rat mandible is preserved, without complete separation of the mandible segments. In contrast, our model is one of the first descriptions of a severely critical defect with a large separation of bone fragments and stabilization with osteosynthesis plates. This model could more faithfully reproduce the clinical situation in which patients and surgeons are confronted with severe trauma, tumors, congenital defects, and other severe conditions. However, regeneration at the bone defect could be significantly more challenging in our model due to the size and structure of the defect, along with the amount of normal bone tissue surrounding the defect. In this study, we used an HTEB substitute containing human cells pre-differentiated to the osteoblastic cell lineage using previously described induction methods. Despite the fact that these methods are broadly used to induce osteogenic differentiation, and are known to be highly efficient [25], future studies should analyze the phenotype of these cells using enzymatic methods, such as the alkaline phosphatase (ALP) activity assay [37].

Despite these limitations, we found a positive effect of the HTEB implant as compared to controls. First, our results suggest that nanostructured fibrin-agarose grafts may contribute to improvement of the development and function of maxillofacial bones as determined by morphological analysis. In fact, bite function, tooth morphology, and facial symmetry tended to be more physiological in animals grafted with these biomaterials as compared to N-CTR animals. Although these results are preliminary, our results suggest that this technology could be useful in cases with severe morphological and structural alterations with loss of mandibular bone. Even though they used a different animal model, previous works carried out by our research group demonstrated that fibrin-agarose-based bioartificial tissues could contribute to induce harmonic development of maxillofacial structures in rabbits with a palate bone defect [26,38].

Compared with other biomaterials used in tissue engineering of the mandibular bone, fibrin-agarose offers high biocompatibility, although its biomechanical properties are not comparable to those of native bone [29]. Among the biomaterials used in mandibular bone tissue engineering, most reports make use of different types of inorganic salts—especially calcium phosphate, alone or combined with growth factors and mesenchymal stem cells [10,23]. Despite the fact that these biomaterials can improve bone formation, their real utility in severely critical defects in which a large percentage of the mandible is lost is very low, and novel strategies based on tissue engineering and cell differentiation are needed in order to efficiently treat these conditions. In general, the fibrin-agarose biomaterial used in the present work offers high biocompatibility to cells cultured within the biomaterial, along with excellent biointegration at the graft site once grafted in vivo.

In the present work, we found that the tissue generated at the defect site varied between groups although, unfortunately, this tissue was not compatible with dense, normal bone tissue in any of the study groups. Interestingly, we found that the use of a bone substitute in the HTEB group was able to improve regeneration of the bone defect as compared to the N-CTR group, and this improvement was associated with structural and histological differences from controls. Regarding the regeneration tissue found at the defect site, we found significant differences in terms of CT radiodensity, with HTEB resulting in the formation of denser tissue as compared to N-CTR and ACELL, although the radiodensity found in P-CTR normal bone was not reached. In general, we could conclude that the use of a fibrin-agarose bioartificial tissue was able to improve tissue regeneration, although regeneration was not comparable to that of native bone, and further research is needed in order to find a perfect bone substitute for this severely critical bone defect model.

When the regeneration tissue was analyzed histologically, we found several differences between groups. Interestingly, animals treated with the fibrin-agarose biomaterial showed some areas of bone formation, while HTEB animals also had detectable areas of cartilage tissue. The structure and composition of these tissues revealed that the regenerating bone corresponding to HTEB was more similar to normal control bone than the regenerating tissue found in other study groups. In short, the bone areas formed in the HTEB animals had similar staining signals to native positive controls for alizarin red and versican, suggesting that this bone could be partially mineralized, and could be associated with this important proteoglycan with a key function in ECM physiology [39]. In addition, HTEB showed significantly higher staining signals than N-CTR for alizarin red, picrosirius red, versican, and osteocalcin. Regarding the first of these markers, our results highlight the possibility that the ECM of the bone regeneration tissue formed in HTEB animals could be more physiological than that found in negative controls. In addition, the higher presence of osteocalcin—considered to be the most specific marker for osteoblastic activity [40]—suggests that the osteoblastic activity of cells found at the regeneration site was more intense in the HTEB group, with signal intensity comparable to, or even higher than, positive controls. This could be explained by the fact that the bone regenerative and biosynthetic activity was more active in HTEB animals than in native controls, which usually display a basal metabolic activity. Interestingly, the osteoid activity as determined by toluidine blue was also higher in the HTEB group than in native, normal controls, suggesting again that this newly formed bone could be related to an active process of biosynthetic activity [41].

Though these findings suggest a partially positive effect of the HTEB on mandibular bone formation, it is clear that the product could be significantly improved to achieve full bone regeneration. On the one hand, novel biofabrication methods should be applied to the HTEB in order to generate a product with higher analogy to the native mandible bone. In this regard, previous reports from our research group demonstrate that fibrin-agarose biomaterials can be improved and functionalized by chemical cross-linking [42], or by combination with magnetic nanoparticles [43] and bioactive nanostructured lipid carriers [44]. Future works should determine whether these methods can enhance the biological and biomechanical properties of the HTEB used in the present work. On the other hand, a crucial step in the development of novel therapies for use in regenerative medicine is clinical translation. As is the case of previous human tissues generated with nanostructured fibrin-agarose biomaterials [17,45], HTEB will need to fulfill several requirements of medicines agencies before therapeutic use in patients.

## 5. Conclusions

In conclusion, the present study is one of the first descriptions of a severely critical mandibular bone defect animal model that could reproduce severe clinical conditions. This model allowed us to evaluate a novel model of tissue-engineered bone substitute that showed positive outcomes in terms of facial bones’ morphology and function and, partially, in terms of regeneration tissue density, structure, and mineralization. Future studies should be carried out in order to determine the usefulness of this bone substitute in different experimental settings.

## Data Availability

The data presented in this study are available upon request from the corresponding author.
